# Supplementary material for: V-CARE (Virtual Care After REsuscitation): Protocol for a Randomized Feasibility Study of a Virtual Psychoeducational Intervention After Cardiac Arrest—A STEPCARE Sub-Study
Source: J Clin Med. 2025 Jun 22;14(13):4429. doi: 10.3390/jcm14134429 (PMC12249823; doi:10.3390/jcm14134429)
Supplement: Supplementary file 1 [file jcm-14-04429-s001.zip › jcm-3651094-supplementary.pdf]

Table S1 - Thematic analysis of data from focus groups with cardiac arrest survivors and their partners – table of themes, subthemes, and codes

Themes	Subthemes and corresponding codes	Supporting quotes
**Theme one:**<br><br>**Life after cardiac arrest–difficulties and challenges**	***1.1 Overwhelmed and under-supported***<br><br>- Misinformed<br>- Feeling let down<br>- Overwhelmed with information<br>- Not enough information/advice<br>- Disregarded<br>- Let down by professionals<br>- Lack of consistency or 'point person'<br>- Isolated and alone<br>- Neglected	- *I wasn't given any details of cardiac rehab or really any details of what to do afterwards.*<br>- *In the early days, you're just overwhelmed. You get told by your doctor you're getting an ICD, here's a leaflet from the makers of the ICD, off you go. You're just overwhelmed. Too much information.*<br>- *I knew nothing about cardiac arrest. I thought a cardiac arrest and a heart attack were the same thing.*<br>- *I got sent home without any leaflets, no pieces of paper, no conversation.*<br>- *We managed like that for six months. We were terrified. We didn't know what to expect. There seemed to be nobody.*<br>- *There's this lovely handbook but there's pages and pages and pages of it. To wade all through that…*
	**1.2 Progressing physically, but not mentally**<br><br>- Change in emotional status<br>- Mental health deterioration<br>- Memory difficulties<br>- Fatigue<br>- Survivor guilt	- *At the moment still struggle with my emotions I think and memory. They're my two killers.*<br>- *I thought I had dementia because I couldn't remember anything. I couldn't read and retain anything. It was just bizarre.*<br>- *(Name's) mental health deteriorated massively after that. The best way to describe it is she couldn't be alone and that continued for at least six months. She couldn't move off the chair in case it happened again.*<br>- *The medical people were really interested in the functional part of the human brain but not the emotional part. There seemed to be a huge detachment from that. That was a huge difficulty to overcome.*<br>- *It wasn't scary for me; it was more for my husband who brought this person he didn't know home. I was not myself at all.*

	**1.3 Difficulty moving on with life**<br>- Identity crisis – "not me/them"<br>- Life is different<br>- Living life in fear<br>- Disconnected from partner<br>- What is the new normal	- *My husband didn't sleep he said for 6 months. He thought it was going to happen every night again. I slept fine. I was completely oblivious.*
**Theme two: Moving forward – what helped me the most**	***2.1 Finding a "point person"***<br>- Consistent support person<br>- Feeling cared for/about<br>- Someone to talk to<br>- Personable and approachable<br>- There when needed<br><br>***2.2 Learning and making sense of things***<br>- Thirst for knowledge /information<br>- Practical advice<br>- Signposting<br>- Filling in the gaps<br>- Making sense of things	- *She made the world of difference. She came out to see us. She spent a couple of hours with us. She moved his meds around because he was having a really terrible time with his medication.*<br>- *Unintentionally this fabulous nurse just took it on herself to make sure we were ok.*<br>- *Just to talk to somebody instead of having to google what a cardiac arrest was, why it was different to a heart attack.*<br><br>- *All these practical things, it would be a great to be able to have buddy sessions or something else about that. Or the driving licence fiascos and how to fill those forms in before you do it wrong.*<br>- *I probably started my PHD in hearts when (name) was in ICU. I just studied and studied and studied.*<br>- *There's that thirst for information. For me, that helped me cope and understand. I needed to understand.*<br>- *To understand terminology in layman's terms.*<br>- *I think it's just knowledge and knowing it's ok to feel the way that you feel, whatever that might be.*<br>- *Making sure people understand what's happened to them, and their family understands what's happened to them and they are going to get better.*<br><br>- *You want peer support and to understand that what you're going through is*

	2.3 Sharing experiences and connecting with others<br><br>- Normalising it<br>- I'm not alone<br>- Peer support<br>- Feeling connected<br>- Telling my story	*normal. That you're not alone.*<br>- *I would have liked to talk to a group of people who were family members, who were rescuers that had come across the scene that we came across to normalise it as you said.*<br>- *When you meet people, you get it, you get an instant connection*<br>- *The first time we met, they were on a different level. They were talking about different experiences. It definitely helps for those involved to be able to just talk to each other. They've got completely different emotions to us.*
**Theme three: A virtual support group – key considerations and recommendation s**	**3.1 Structure and size**<br><br>- Intimate but not too intimate<br>- Non-threatening<br>- One hour max<br>- Managing domineering individuals<br>- Risk of passive passengers<br>- Separate groups for partners/survivors	- *We've got about 8 or 10 people on the call tonight. That feels intimate enough that you can say what you want to say, answer your questions and hopefully other people say things you wouldn't have thought about.*<br>- *Apparently, my wife opened up but she hadn't opened up when I was in the room. I think a separate session would be very useful.*<br>- *There was about 20 people plus and then you had the vocal three so everyone else just became a passive passenger. You know, you lost a lot in there. This is a nice number here.*<br>- *To ask questions that he wasn't party to, so that I could say I'm really really concerned about this. I didn't want to say in front of him because it would have worried him more.*<br>- *Basically I switched off towards the end and although I had questions I didn't ask them because after an hour and a half I'd had enough and just wanted to get out of the room because I was tired.*<br>- *Must be a time for questions and I think an hour is sufficient.*<br><br>- *I like the idea of the first one being a more open-ended question and answer.*

	**3.2 Suggested content**<br><br>- General introduction<br>- Unstructured<br>- Chance to tell my story<br>- Practical advice<br>- First hand experiences<br>- Signposting<br>- Emotions<br>- Cognitive changes<br>- Relationships<br>- Going back to work<br>- Supporting written materials	*We all got information overloaded at the beginning.*<br>- *Early on you should have something that signposts to some of the information on SCA, on the Facebook group*<br>- *Presentation of information but also people being able to connect. Even just at the beginning of the session people can just say where they're at, you know, and just be heard, and be like yeh we get it, we hear you.*<br>- *I think there's something really important to have some written information as well. Almost like, you know, there's the booklet that goes with the sessions. I find it really hard to retain certain types of information and things verbal communicated were just mush.*<br>- You do need a key session on the practicalities. I better not have my mobile phone in that pocket, I better not had my watch on that side… those sorts of things.
	**3.3. Finding the right facilitator**<br><br>- Non-threatening<br>- Someone who gets it<br>- Personable<br>- Rotate weekly<br>- Range of experts<br>- Survivor also present	- *I didn't need somebody who was like an expert or a consultant, I just needed someone who actually knew what they were talking about on an on the ground type level.*<br>- *Not so much a specialist but someone who's actually been there. Agree with you that cardiac nurses know it all, CHS nurses, they were very very helpful. They weren't experts but they knew exactly what we were going through and how to get the best out of us. Just putting your mind at rest so it's more personable.*<br>- *Some people may be intimidated by having a medical person there because there's something they don't want to share.*<br>- *What I'm thinking is to look at different facilitators so you've got various aspects of the medical side but for survivors you've also got another survivor, for example. For life saver's, another life saver. Maybe someone from the ambulance service, a call taker. Maybe a first responder. Maybe someone from the voluntary service.*
		- *I think it needs to have a number of options. None of us are the same we are all individuals going through an individual process. I don't think a one size fits all is*

	**3.4 Avoiding a one-size fits all approach**<br><br>- Catering for different learning styles<br><br>- Flexibility<br><br>- Different needs for different stages<br><br>- Dip in, dip out	*the way ahead.*<br>- *What the physio group did is they ran a series of six sessions on things like healthy eating, on medication, whatever, but they didn't have to attend all of them. If they missed it on one set of six, they were able to come in on the next one.*<br>- *I don't know if there's a way of these are the broad topics, people have an option to 'yeh I'm ready for those two sessions but I'm not quite ready for that'*<br>- *Giving yourself a suite of options. You could have workshops. You could have peer-to-peer reviews. 1-1 with medical people, 1-1 with psychologists.*

TIDieR

V-CARE (Virtual Care After REsuscitation) trial – a comparative, single-blind randomized sub-study of the STEPCARE trial.

**Why:**	OHCA survivors and their key supporters (family/friends) face ongoing challenges post-discharge, including psychological distress, fatigue, cognitive issues, and difficulty returning to daily activities. There is limited evidence on feasible, scalable post-ROSC psychoeducational interventions. V-CARE aims to assess the feasibility and acceptability of a brief, remotely delivered group intervention and inform future development of effective post-OHCA recovery support.
**What (material):**	**Intervention group:** Four weekly, semi-structured, one-hour psychoeducational sessions via video call. Materials include PowerPoint slides covering:<br>1. Understanding cardiac arrest<br>2. Coping with fatigue and memory problems<br>3. Managing low mood and anxiety<br>4. Returning to daily life<br>**Control group:** A 9-page digital information booklet co-developed with OHCA survivors and supporters, including practical guidance on memory, mood, fatigue, and recovery.
**What (procedures):**	The sessions combine education, peer interaction, and skill-building. Each session aims for a 50/50 split between presentation and discussion. Group size: 4–12 participants. Groups are formed once minimum numbers are met and delivered around 2–3 months post-discharge. The control group receives the digital booklet and completes follow-up one month later.
**Who provided:**	V-CARE sessions are delivered by a trained healthcare professional (Clinical Psychologist, Occupational Therapist, or Clinical Nurse Specialist). A cardiologist or cardiology trainee joins the first session to answer cardiac-specific questions. The control group may contact a study researcher for clarification on booklet content.
**How (mode of delivery; individual or group):**	**V-CARE:** Group sessions delivered remotely via video conferencing software.<br>**Control:** Information booklet provided digitally to each participant.
**Where:**	Participants attend V-CARE sessions from home using a personal device. Control group participants read the booklet at home
**When and how much:**	**V-CARE:** Four sessions, one per week, each approximately 60 minutes.<br>**Booklet:** One-time delivery.
**Tailoring:**	No individualized tailoring, though discussion content may vary slightly due to participant questions and group dynamics.
**How well (planned):**	Fidelity will be monitored using a bespoke rating scale assessing adherence to content and session structure for a sub-set of the sessions (10-20%). Session duration, completion rates, questionnaire completion, and protocol deviations will also be logged.

File S1: V-CARE group intervention - PowerPoint presentations

V-CARE – Group

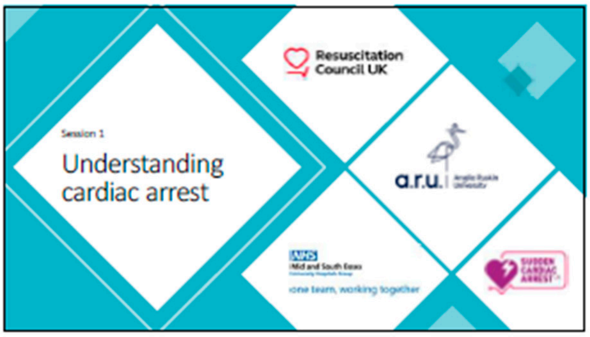

1

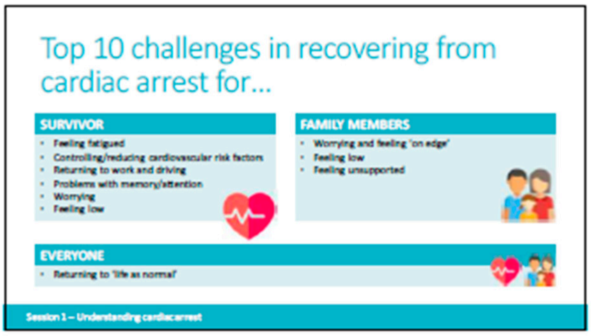

2

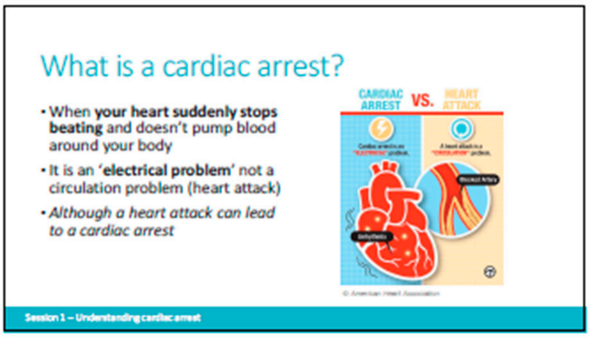

3

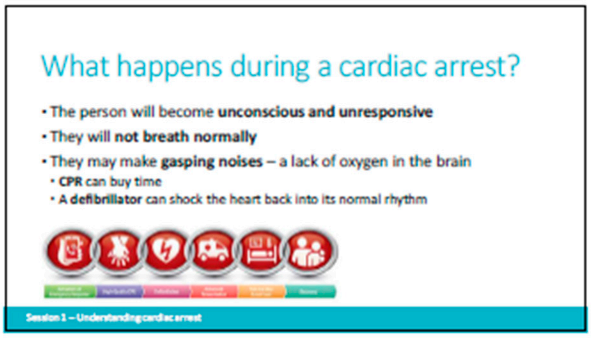

4

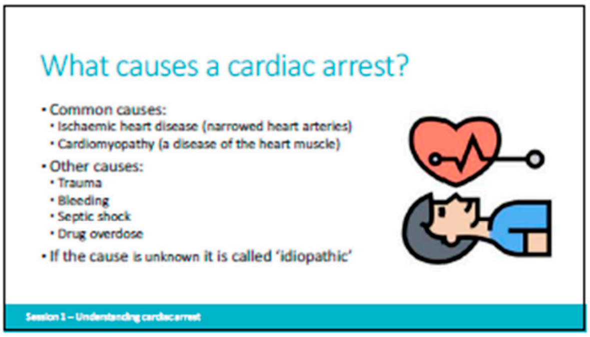

5

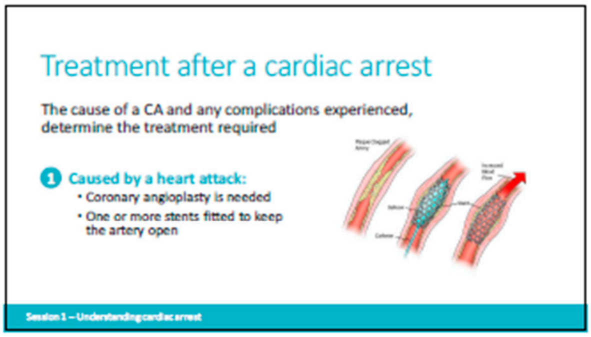

6

Treatment after a cardiac arrest

The cause of a CA and any complications experienced, determine the treatment required

2 Caused by a cardiomyopathy or an unknown cause:
- Fitting an Implantable Cardioverter Defibrillator (ICD) is recommended

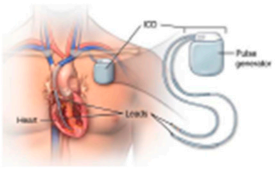

7

Medication

Medication prescribed will depend on the cause of your CA

If caused by a heart attack:
- **Lower your blood pressure** sometimes called 'ACE inhibitors', such as Ramipril
- **Prevent blood clots** 'antiplatelets' Aspirin and a similar medication (i.e. clopidogrel)
- **Beta blockers** Help relax the heart's muscle, and protect it from further damage.
- **Statins** Reduce your cholesterol.

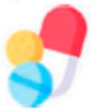

Even if your CA was not caused by a heart attack, you may be prescribed some of the drugs listed here

8

What follow-up should I expect?

Unfortunately, standards of care vary a lot across the country. Follow-up care also depends on your presenting problems and the treatment you received.

Nationwide
- *Intensive care follow-up:* three months after discharge

Purpose of the follow-up appointment
- Discuss any questions about medication and symptoms you're experiencing

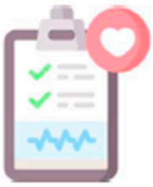

9

Some common issues in recovery – can you relate to some of them?

SURVIVOR
- Feeling tired (fatigued)
- Return to driving
- How much physical exercise is 'too much'
- Feeling worried of having another CA
- Feeling down and anxious
- Wanting / not wanting to change diet or lifestyle
- Lacking confidence to be on your own
- Losing confidence in your own abilities
- Not remembering the cardiac arrest
- Having difficulties with memory and attention

FAMILY MEMBER
- Difficulty sleeping and relaxing
- Experience the event completely different from the survivor, which can cause frustration
- Dealing with changes in temperament / personality
- Feeling 'snappy' and irritable
- Feeling constantly 'on guard', and anxious
- as if something bad could happen
- Feeling traumatised by the whole event

10

Can I prevent this happening again?

FACTORS YOU CAN CONTROL
- High blood pressure
- High cholesterol
- Diabetes
- Smoking
- Being overweight
- Amount of physical activity

FACTORS YOU CAN'T CONTROL
- Family history (genetics)
- Age
- Ethnic background

11

FAQs

So, what can I do to stop it from happening again?

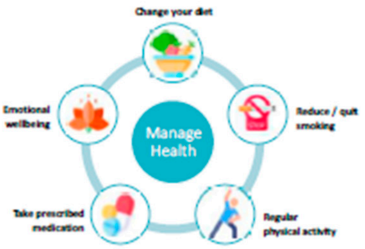

12

FAQs

How much exercise should I do?

You might get tired more easily now…

1. Cardiac rehabilitation
2. Walk more
3. Don't strain to lift anything

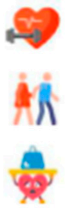

Session 1 – Understanding cardiac arrest

13

FAQs

I don't remember anything of my CA, including a few hours/days before and after it. Is this normal?

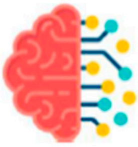

Yes, this is normal.

Some people remember everything up to the moment of their cardiac arrest, others have no memories.

Session 1 – Understanding cardiac arrest

14

FAQs

My husband/wife has just had a cardiac arrest. I feel very nervous when I see him/her go out on their own as I am worried it might happen again.

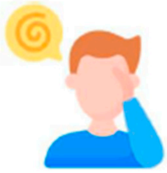

This feeling is very normal.

Most people find they become less nervous as time goes on, especially if they build up their tolerance little by little

Session 1 – Understanding cardiac arrest

15

FAQs

You are likely to have many more questions on medication, secondary prevention, physical activity etc.

Please feel free to ask them now!

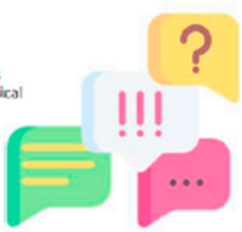

Session 1 – Understanding cardiac arrest

16

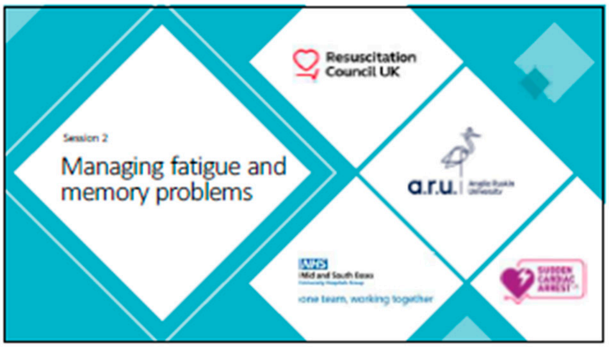
Resuscitation Council UK
Session 2
Managing fatigue and memory problems
a.r.u.
one team, working together
SUDDEN CARDIAC ARREST

1

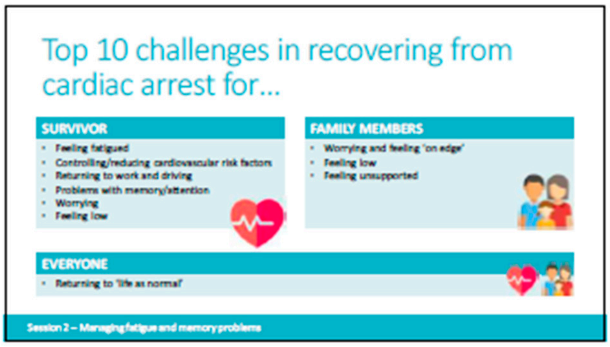
Top 10 challenges in recovering from cardiac arrest for…
SURVIVOR
Feeling fatigued
Controlling/reducing cardiovascular risk factors
Returning to work and driving
Problems with memory/attention
Worrying
Feeling low
FAMILY MEMBERS
Worrying and feeling 'on edge'
Feeling low
Feeling unsupported
EVERYONE
Returning to 'life as normal'
Session 2 – Managing fatigue and memory problems

2

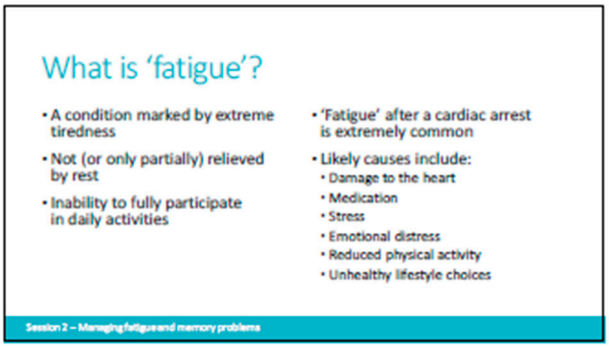
What is 'fatigue'?
A condition marked by extreme tiredness
Not (or only partially) relieved by rest
Inability to fully participate in daily activities
'Fatigue' after a cardiac arrest is extremely common
Likely causes include:
Damage to the heart
Medication
Stress
Emotional distress
Reduced physical activity
Unhealthy lifestyle choices
Session 2 – Managing fatigue and memory problems

3

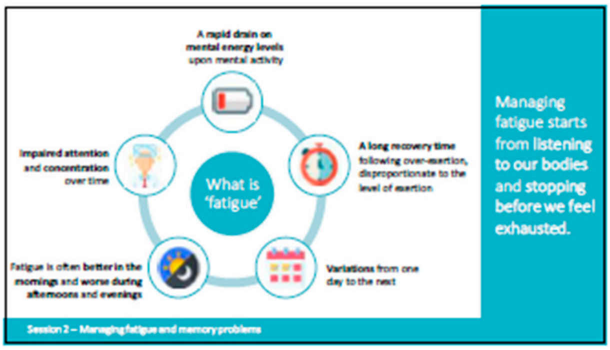
A rapid drain on mental energy levels upon mental activity
Impaired attention and concentration over time
What is 'fatigue'
A long recovery time following over-exertion, disproportionate to the level of exertion
Fatigue is often better in the mornings and worse during afternoons and evenings
Variations from one day to the next
Managing fatigue starts from listening to our bodies and stopping before we feel exhausted.
Session 2 – Managing fatigue and memory problems

4

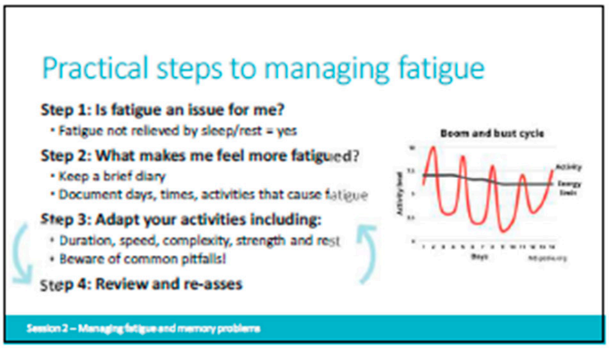
Practical steps to managing fatigue
Step 1: Is fatigue an issue for me?
Fatigue not relieved by sleep/rest = yes
Step 2: What makes me feel more fatigued?
Keep a brief diary
Document days, times, activities that cause fatigue
Step 3: Adapt your activities including:
Duration, speed, complexity, strength and rest
Beware of common pitfalls!
Step 4: Review and re-asses
Boom and bust cycle
Activity
Days
Session 2 – Managing fatigue and memory problems

5

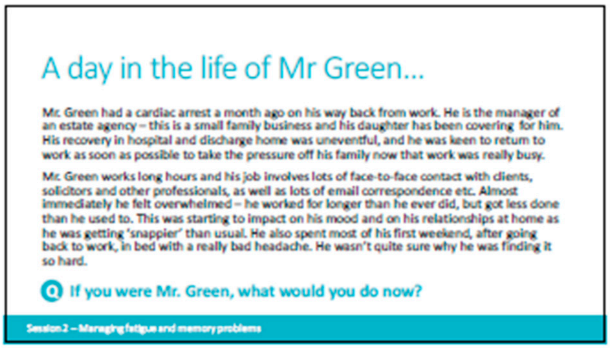
A day in the life of Mr Green…
Mr. Green had a cardiac arrest a month ago on his way back from work. He is the manager of an estate agency – this is a small family business and his daughter has been covering for him. His recovery in hospital and discharge home was uneventful, and he was keen to return to work as soon as possible to take the pressure off his family now that work was really busy.
Mr. Green works long hours and his job involves lots of face-to-face contact with clients, solicitors and other professionals, as well as lots of email correspondence etc. Almost immediately he felt overwhelmed – he worked for longer than he ever did, but got less done than he used to. This was starting to impact on his mood and on his relationships at home as he was getting 'snappier' than usual. He also spent most of his first weekend, after going back to work, in bed with a really bad headache. He wasn't quite sure why he was finding it so hard.
Q If you were Mr. Green, what would you do now?
Session 2 – Managing fatigue and memory problems

6

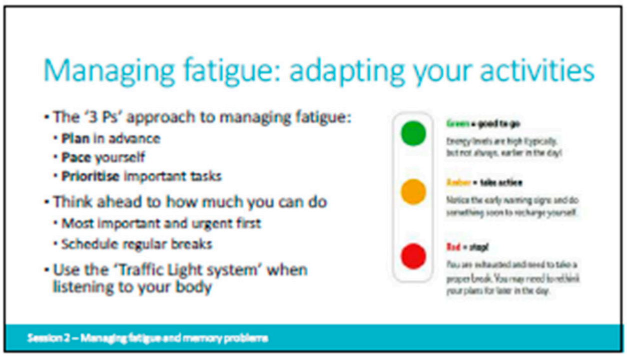
Managing fatigue: adapting your activities
The '3 Ps' approach to managing fatigue:
Plan in advance
Pace yourself
Prioritise important tasks
Think ahead to how much you can do
Most important and urgent first
Schedule regular breaks
Use the 'Traffic Light system' when listening to your body
Green = good to go
Energy levels are high typically, but not always, earlier in the day!
Amber = take action
Notice the early warning signs and do something soon to recharge yourself.
Red = stop!
You are exhausted and need to take a proper break. You may need to rethink your plans for later in the day.
Session 2 – Managing fatigue and memory problems

7

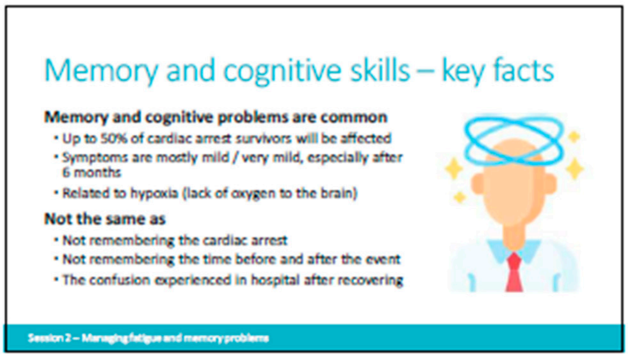
Memory and cognitive skills – key facts
Memory and cognitive problems are common
Up to 50% of cardiac arrest survivors will be affected
Symptoms are mostly mild / very mild, especially after 6 months
Related to hypoxia (lack of oxygen to the brain)
Not the same as
Not remembering the cardiac arrest
Not remembering the time before and after the event
The confusion experienced in hospital after recovering
Session 2 – Managing fatigue and memory problems

8

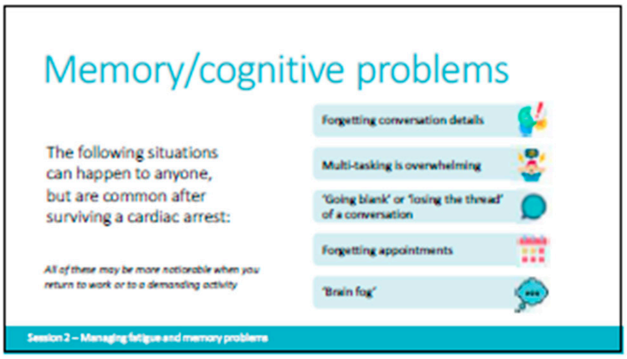
Memory/cognitive problems
The following situations can happen to anyone, but are common after surviving a cardiac arrest:
All of these may be more noticeable when you return to work or to a demanding activity
Forgetting conversation details
Multi-tasking is overwhelming
'Going blank' or 'losing the thread' of a conversation
Forgetting appointments
'Brain fog'
Session 2 – Managing fatigue and memory problems

9

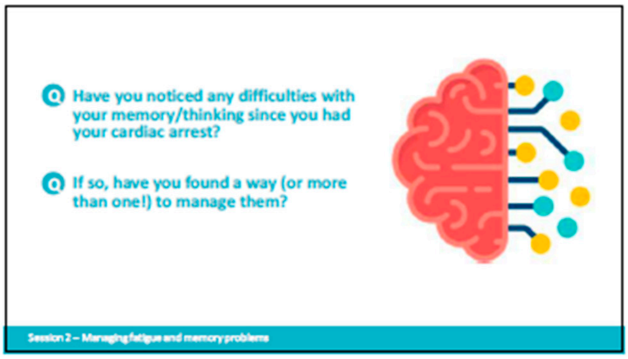
Have you noticed any difficulties with your memory/thinking since you had your cardiac arrest?
If so, have you found a way (or more than one!) to manage them?
Session 2 – Managing fatigue and memory problems

10

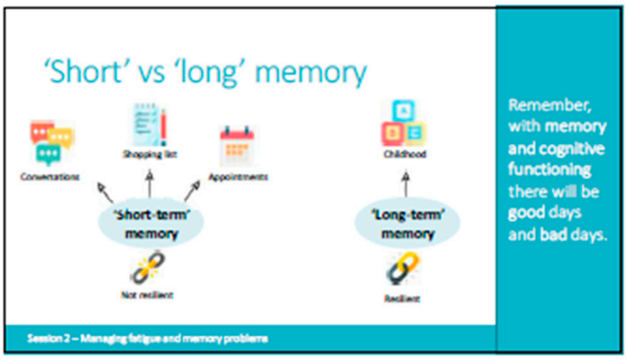
'Short' vs 'long' memory
Conversations
Shopping list
Appointments
'Short-term' memory
Not resilient
Childhood
'Long-term' memory
Resilient
Remember, with memory and cognitive functioning there will be good days and bad days.
Session 2 – Managing fatigue and memory problems

11

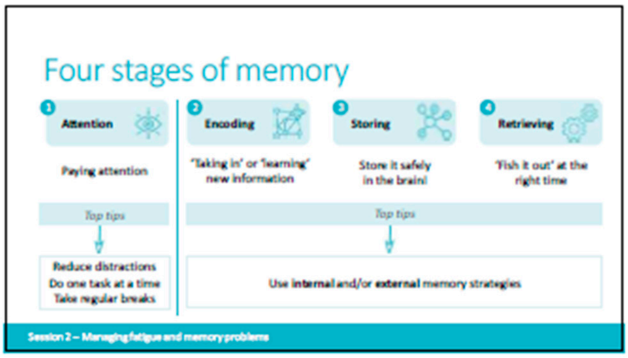
Four stages of memory
1 Attention
Paying attention
2 Encoding
'Taking in' or 'learning' new information
3 Storing
Store it safely in the brain!
4 Retrieving
'Fish it out' at the right time
Top tips
Reduce distractions
Do one task at a time
Take regular breaks
Top tips
Use internal and/or external memory strategies
Session 2 – Managing fatigue and memory problems

12

Two broad types of strategies

INTERNAL

- **Repetitions**
  - Repeat the same information several times
  - Space out (1min, 5mins, 20mins etc)
- **Chunk** information together
  - e.g. shopping list: ' vegetables', 'drinks', etc
- **Visualise**
  - Create a mental image of what you want to remember

Worth a try, but maybe less likely to work

EXTERNAL

- Planners and calendars
- Whiteboards
- Post-it notes
- Take photographs and/or notes
- Always put objects back in the same place
- Electronic reminders (smartphones, tablets)
- Medication blister packs or dosette boxes

More reliable and practical (if used correctly!)

13

Less common cognitive problems

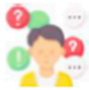

Struggling to the find the right words?

- Due to reduced attention / high levels of fatigue
- Avoid getting extremely fatigued
- Avoid getting too frustrated

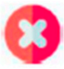

Feeling unmotivated?

- Due to low mood
- Due to effect of the cardiac arrest on the brain
- Keep a daily / weekly routine

14

FAQs

Do the memory problems get better with time?

Yes, they usually get better.

Most of the time they don't get any worse, nor are they an indication you might have dementia.

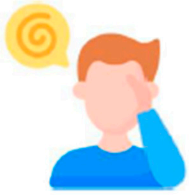

15

FAQs

I'm about to change job/house/country/partner etc – should I continue?

This is entirely your choice.

However waiting a few weeks allows for any initial problems to settle down (e.g. confusion, memory difficulties, anxiety, low mood etc)

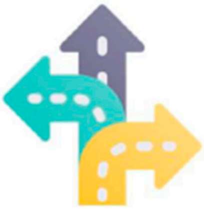

16

FAQs

You are likely to have many more questions on managing fatigue and memory difficulties...

Please feel free to ask them now!

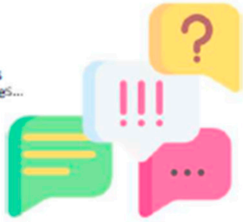

17

Session 3

Understanding and managing your mood

Resuscitation Council UK

a.r.u. Anglia Ruskin University

NHS Mid and South Essex University Hospitals Group

one team, working together

Sudden Cardiac Arrest

1

Top 10 challenges in recovering from cardiac arrest for…

SURVIVOR

- Feeling fatigued
- Controlling/reducing cardiovascular risk factors
- Returning to work and driving
- Problems with memory/attention
- Worrying
- Feeling low

FAMILY MEMBERS

- Worrying and feeling 'on edge'
- Feeling low
- Feeling unsupported

EVERYONE

- Returning to 'life as normal'

Session 3 – Understanding and managing your mood

2

Feeling low and anxious – is it common?

Lots of studies have looked into this…

- Anxiety, depression and PTSD following cardiac arrest: A systematic review of the literature
- Health-related quality of life after surviving an out-of-hospital compared to an in-hospital cardiac arrest: A Swedish population-base registry study
- Psychological wellbeing in survivors and its relationship to neurocognitive function
- One-year longitudinal study of psychological distress and self-assessed health in survivors of out-of-hospital cardiac arrest

…and it is fairly common (around 1 in 4)

Session 3 – Understanding and managing your mood

3

Feeling low and anxious – is it common?

Equally/more common in relatives/family

- Feeling responsible but unsupported: How relatives of out-of-hospital cardiac arrest survivors experience the transition from hospital to daily life – A focus group study
- Long-term quality of life of caregivers of cardiac arrest survivors and the impact of witnessing a cardiac event of a close relative
- Relatives' experiences of everyday life six months after hypothermia treatment of a significant other's cardiac arrest
- Trapped in a disrupted normality: Survivors' and partner' experiences of life after a sudden cardiac arrest
- Life after cardiac arrest: The importance of engaging with the 'forgotten patient'

…this is also very common (1 in 4 or more)

Session 3 – Understanding and managing your mood

4

Q What sort of psychological challenges do you think are common after surviving a cardiac arrest?

Q What challenges do you think family members face?

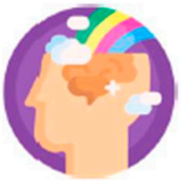

Session 3 – Understanding and managing your mood

5

Challenges after a cardiac arrest

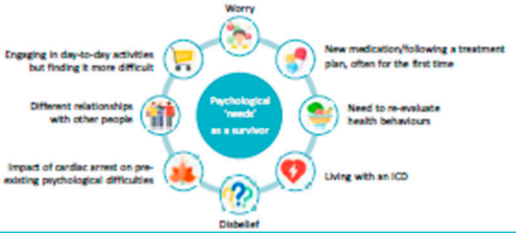

Session 3 – Understanding and managing your mood

6

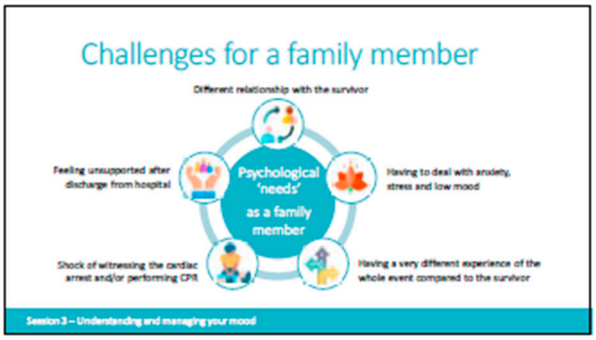
Challenges for a family member
Different relationship with the survivor
Feeling unsupported after discharge from hospital
Psychological 'needs' as a family member
Having to deal with anxiety, stress and low mood
Shock of witnessing the cardiac arrest and/or performing CPR
Having a very different experience of the whole event compared to the survivor
Session 3 – Understanding and managing your mood

7

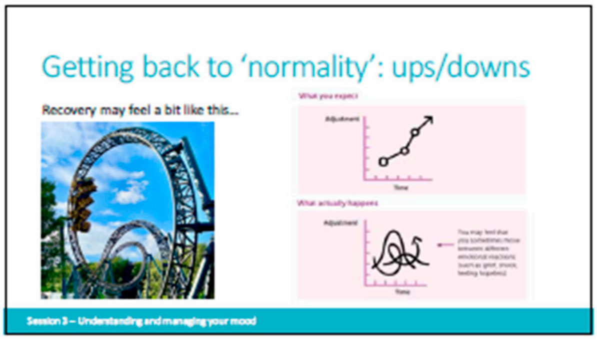
Getting back to 'normality': ups/downs
Recovery may feel a bit like this…
What you expect
Adjustment
Time
What actually happens
Adjustment
Time
Session 3 – Understanding and managing your mood

8

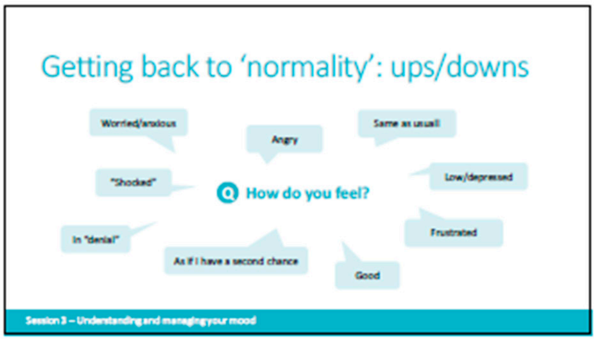
Getting back to 'normality': ups/downs
Worried/anxious
Angry
Same as usual!
"Shocked"
How do you feel?
Low/depressed
In "denial"
Frustrated
As if I have a second chance
Good
Session 3 – Understanding and managing your mood

9

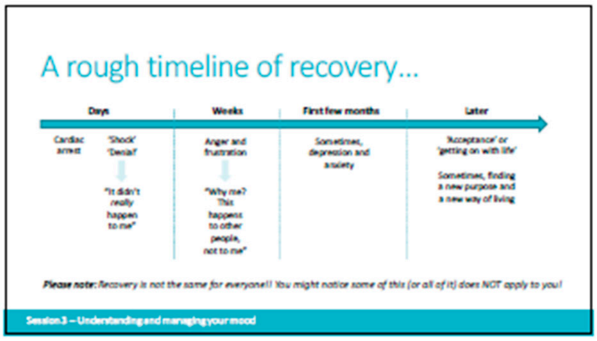
A rough timeline of recovery…
Days
Weeks
First few months
Later
Cardiac arrest
'Shock' 'Denial'
"It didn't really happen to me"
Anger and frustration
"Why me? This happens to other people, not to me"
Sometimes, depression and anxiety
'Acceptance' or 'getting on with life'
Sometimes, finding a new purpose and a new way of living
Please note: Recovery is not the same for everyone!! You might notice some of this (or all of it) does NOT apply to you!
Session 3 – Understanding and managing your mood

10

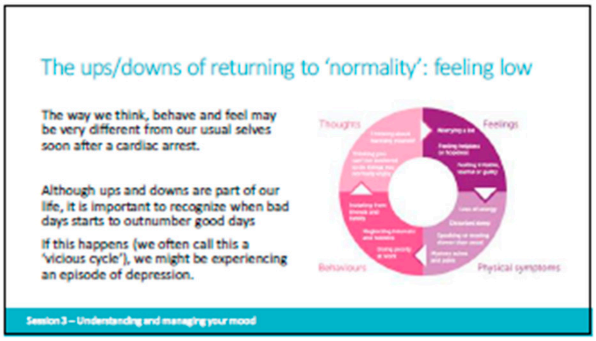
The ups/downs of returning to 'normality': feeling low
The way we think, behave and feel may be very different from our usual selves soon after a cardiac arrest.
Although ups and downs are part of our life, it is important to recognize when bad days starts to outnumber good days
If this happens (we often call this a 'vicious cycle'), we might be experiencing an episode of depression.
Thoughts
Feelings
Behaviours
Physical symptoms
Session 3 – Understanding and managing your mood

11

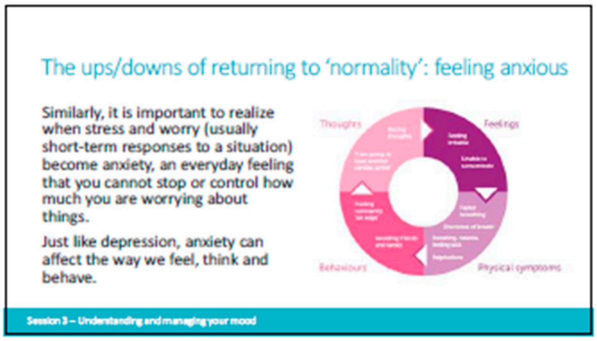
The ups/downs of returning to 'normality': feeling anxious
Similarly, it is important to realize when stress and worry (usually short-term responses to a situation) become anxiety, an everyday feeling that you cannot stop or control how much you are worrying about things.
Just like depression, anxiety can affect the way we feel, think and behave.
Thoughts
Feelings
Behaviours
Physical symptoms
Session 3 – Understanding and managing your mood

12

'Adjustment' for a survivor

My world = my beliefs and assumptions	Cardiac arrest	Challenge my beliefs and assumptions	Progressive adjustment to changes
'I'm invincible'; 'Other people have heart problems, not me.'	'OUCH'	'Maybe I am not invincible, maybe I need to change something, or look at things in a different way'	'While I keep recovering, I can do things a bit differently. Or do a bit less. That's ok.'
'I can do all my things without problems.'		'Some things are a bit harder now. I tire easily. Not yet back to 100%.'	

Session 3 – Understanding and managing your mood

13

'Adjustment' for a family member

My world = my beliefs and assumptions	Cardiac arrest	Challenge my beliefs and assumptions	Progressive adjustment to changes
'Heart problems happen to other people.'	'OUCH'	'Are they going to be safe on their own? '	'We can cope. Things are slowly getting better.'
		'Are we going to be ok?'	
		'What about all our plans?'	

Session 3 – Understanding and managing your mood

14

Wellbeing: what helps

Keep active

- Do something every day, no matter how small.
- Make a plan for the week ahead.
- Include activities that give you a sense of pleasure/achievement.
- If you are able, go for a walk, spend sometime outside, talk to a friend/family member

Health and wellbeing

- Think of quitting smoking
- Think of losing weight
- Drink less alcohol
- Get good quality sleep
- Manage your stress levels

Be kind to yourself

- Very strong beliefs can become an obstacle.
- Having to make changes is not a sign of failure. It often is a sign of growth.
- Many people find a renewed sense of personal growth and confidence after a cardiac arrest.

Session 3 – Understanding and managing your mood

15

FAQs

Is mood and/or anxiety affecting you?/ How?

After this session, think of one or two strategies/changes you could make to manage this better.

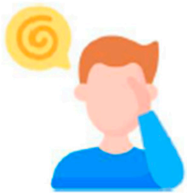

Session 3 – Understanding and managing your mood

16

Further help

As we always say.....if you are struggling
(or if you find yourself struggling in the future...)

You are **Not Alone**

Cardiac Arrest Peer Support

Lots of support is available:

Peer support
(i.e. the people who just 'get it' and know what you are talking about):

- https://www.suddencardiacarrestuk.org/
- https://www.facebook.com/SuddenCardiacArrestUK
- https://twitter.com/WeAreSCAUK

Access support via the NHS:

- https://www.nhs.uk/mental-health/

Other services offering support:

- https://www.mind.org.uk/

Session 3 – Understanding and managing your mood

17

Session 4

All the 'practical stuff' of day-to-day life

Resuscitation Council UK

a.r.u. Anglia Ruskin University

NHS Mid and South Essex Community Hospitals Group

one team, working together

Sudden Cardiac Arrest

1

Top 10 challenges in recovering from cardiac arrest for…

SURVIVOR

- Feeling fatigued
- Controlling/reducing cardiovascular risk factors
- Returning to work and driving
- Problems with memory/attention
- Worrying
- Feeling low

FAMILY MEMBERS

- Worrying and feeling 'on edge'
- Feeling low
- Feeling unsupported

EVERYONE

- Returning to 'life as normal'

2

Driving after a cardiac arrest

Q So, I've had a cardiac arrest. Do I need to stop driving?

- The simple answer is yes, but usually only temporarily.
- The length of time you can't drive is dependent on:
  1. the **cause** of your cardiac arrest
  2. the **remedial action** taken

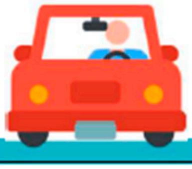

3

Driving after a cardiac arrest

Example #1:

Cause: Heart attack

Remedial action: Stents and medications

Outcome: Stop driving for 4 weeks* (starting when remedial work was done)

If your medications change, you may need to stop driving for 1 month.

**Different rules apply if you have a bus, coach or lorry licence, visit:*

https://www.gov.uk/heart-attacks-and-driving

Example #2:

Remedial action: If you have an ICD fitted

Outcome: Stop driving for 6 months

Check the DVLA regarding assessing your fitness to drive:

https://www.gov.uk/guidance/assessing-fitness-to-drive-a-guide-for-medical-professionals)

4

Driving after a cardiac arrest

Q Do I need to inform the DVLA?

- Yes, it is your responsibility to notify DVLA about a health or medical condition that could affect your driving
- **You can be fined up to £1,000 if you don't**
- Do this via *'Report a medical condition page'*

Note:

- You'll need to enter details about your current driving licence and your condition.
- You may also need your GP's name and address
- You'll need to select from a long list of conditions that may affect your ability to drive.
- Cardiac arrest is NOT listed as a condition (it is an event!)

5

Driving after a cardiac arrest

Q Do I have to inform my insurance company?

- Yes, you should inform your insurance company of your heart condition and implant (ICD/pacemaker) if you have one

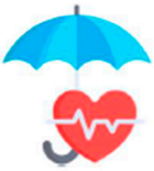

6

Driving after a cardiac arrest

Q I surrendered my driving licence on the advice of my doctor. The period of voluntary surrender has elapsed and I want to reapply.

How do I do that?

- Once the voluntary surrender time has elapsed you are eligible to reapply
- **It's worth starting the process 8 weeks before the restriction ends!**
- To begin the process, visit:

https://www.gov.uk/reapply-driving-licence-medical-condition

- DVLA will send forms to your cardiologist with an expected 6 week turnaround.
- Once your cardiologist has signed these forms, DVLA should issue you with your licence
- If your licence was revoked, the process is likely to take much longer

7

Driving after a cardiac arrest

Q I have an ICD. What happens if I have a shock? Can I still drive?

If you have any shocks, further suspensions will apply, depending on a number of factors. For example:

1. **Receive an appropriate shock:** Surrender your licence. Stop driving for 6 months
2. **Receive an appropriate shock and become unconscious:** 2 year driving suspension
3. **Receive an inappropriate shock:** 1 month driving suspension

If you are driving whilst you receive a shock, stop as soon as it's safe. Do not continue until the cause of the shock has been investigated

8

Returning to work after a cardiac arrest

Key facts:

- 75% Most people return to work
- Some people retire. (Mean age of SCA in the UK is 66 years)
- Some people want to/have to change job or cut hours

- Sudden Cardiac Arrest can put a survivor and their family into a financial crisis – if the survivor provided the main source of income
- A prolonged period of recuperation is not uncommon
- Many people find it necessary to access benefits for a limited period of time

More info can be found at: https://www.suddencardiacarrestuk.org/information/practical-issues/benefits/

9

Returning to work after a cardiac arrest?

- You may have to temporarily resort to help from the **benefits system**
- There can be a myriad of forms and processes to go through and they are often **complicated and unwieldy**
- It is essential to **get advice** and guidance before embarking on any applications

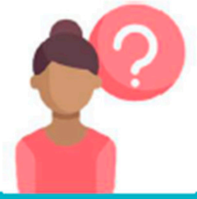

10

Returning to work after a cardiac arrest?

BENEFIT #1

Employment and Support Allowance (ESA)

- Benefit for people with limited capability for work due to sickness or disability, but don't get Statutory Sick Pay
- There are two types of ESA:
  1. *Income-related*
  2. *Contributory*

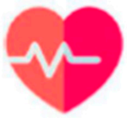

More info here: https://www.gov.uk/employment-support-allowance

11

Returning to work after a cardiac arrest?

BENEFIT #2

Disability Living Allowance (DLA)*

- Benefit for people with extra care needs or mobility needs as a result of a disability
- There are two parts to the DLA. You may qualify for one or both of these:
  1. *Care component*
  2. *Mobility component*

**If you are 16 or over you can no longer make a new claim for DLA*

More info here: https://www.gov.uk/dla-disability-living-allowance-benefit

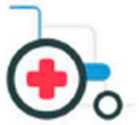

12

Returning to work after a cardiac arrest?

BENEFIT #3

Personal Independence Allowance (PIP)
- Benefit that helps with the extra costs of long-term health condition or disability
- For people aged 16-64
- It's gradually replacing the DLA benefit

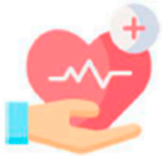

More info here: https://www.gov.uk/dla-disability-living-allowance-benefit

13

Returning to work after a cardiac arrest?

How to cope: 7 top tips

1. Be honest with your employer and colleagues
2. Build up physical and mental activity gradually
3. Be realistic about how you are getting on
4. Manage unexpected tiredness. Avoid stress and get enough sleep
5. If physical requirements are too demanding, seek alternative duties
6. Consider retraining or changing occupation if you can't continue in your previous employment
7. For mentally demanding or stressful jobs learn stress/time-management skills

14

Returning to work after a cardiac arrest?

Survivors' experiences

"Go when u feel your ready as long as your doctor says it's ok. We all heal at different speeds but make sure you're both physically and mentally ok"
Nic

"It takes as long as it takes. Different people have different experiences, give yourself enough time to recover. Your body has gone through a major event and needs recovery time"
Willem

"...nurses and physios who run them [cardiac rehab] are best placed to assess your physical capability to work. But your emotional and psychological capability is less predictable and you might find things triggering a reaction at the most unexpected of times"
Brian

15

Living with an ICD

- If you were given an ICD because of your heart condition, it is likely you will have many questions on what you can and cannot do and what you need to be careful about
  - Read your ICD manual
  - Discuss questions with your cardiologist

Can I go through anti-theft detectors/security systems at the airport?

Can I use a welder/chainsaw/ power tool?

Can I use my smartphone in my shirt pocket?

What happens if I get shocked in a swimming pool?

For a list of common ICD-related questions (and some tentative answers), please see:
https://www.suddencardiacarrestuk.org/faq-category/implantable-devices/

16

Travelling after a cardiac arrest

If you've had a cardiac arrest you might be worried about travelling:
- Will you be able to get appropriate insurance?
- Do you have an ICD? Are you worried about how to manage it far from home?

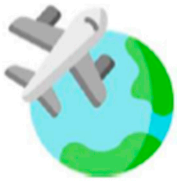

17

Travelling after a cardiac arrest

Travel insurance
- SCA (Sudden Cardiac Arrest) UK members have successfully been able to find appropriate health insurance from a number of different companies. This includes 'elderly' (>65) people with multiple health conditions (in addition to their cardiac arrest), often without a hefty premium. The advice, as always, is to shop around.

SUDDEN CARDIAC ARREST UK

18

Travelling after a cardiac arrest

Travelling with an ICD

Q Are body scanners and security screening equipment safe to use?

- Your ICD is not likely to be affected as the scanning time is short
- Carry your heart device ID card with you. Helpful if your heart device sets off a metal detector or a security system
- Support for your heart device is available around the world
- Your doctor may be able to check your heart device remotely

19

FAQs

You are likely to have many more questions...

Please feel free to ask them now!

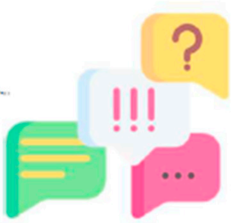

Session 4 – All the 'practical stuff'

20

File S2: V-CARE intervention - digital information booklet

DIGITAL INFORMATION BOOKLET

V-CARE

Dear participant,

This document was developed to inform you about the most common difficulties survivors and their families may experience after a sudden cardiac arrest.

The document is divided into three sections:

Section 1 *Fatigue after a cardiac arrest*

Section 2 *Memory and Cognitive difficulties after a cardiac arrest*

Section 3 *Mental Health and Wellbeing after a cardiac arrest*

We have included some practical tips that we hope both the survivor and their family members will find helpful.

(Dr. …………………………………….)

FATIGUE AFTER CARDIAC ARREST

A cardiac arrest happens when the heart stops beating effectively. It is an 'electrical problem', and as such is different from a heart attack, which is more like a plumbing problem where blood cannot flow properly to parts of the heart. Although a heart attack can sometimes lead to a cardiac arrest, they are not the same thing.

'Fatigue' is a common experience following cardiac arrest; however, there is no universally agreed definition of precisely what this is. It is a personal experience, a bit like pain.

Clinicians agree that persistent fatigue is a feeling of exhaustion which seems disproportionate to our activity levels. Compared to before the Sudden Cardiac Arrest (SCA), fatigue may be more intense, last longer, and not necessarily alleviated by rest.

Fatigue can impact during or after any practical task, from walking and talking to personal care and domestic activities such as meal preparation to managing correspondence, social interactions, recreational activities, and work. It is typically worse after engaging in new/novel activities. Sometimes fatigue is not experienced until some hours later, which makes it challenging to know what has contributed towards it.

Fatigue is not laziness, and because it is one of the 'hidden' challenges following a cardiac arrest, it can go unrecognised as a symptom for some time. It is often not until you are trying to fully go back to life as usual that you become aware of having less mental and physical energy.

WHEN MAY FATIGUE OCCUR?

- Following mental (thinking), physical, or emotional activities, e.g. dealing with correspondence, completing forms, conversations, childcare.
- When in a multi-sensory or group environment, e.g. attending a sporting event, supermarket, restaurant, pub, travelling
- When we find it hard to 'switch our head off' from attempts to problem solve or think things through, which also can impact our sleep, creating a vicious cycle

After disrupted sleep which can contribute to daytime tiredness

Due to insufficient or irregular nutrition/hydration.

When we become unwell

WHAT CAN I DO TO MANAGE FATIGUE?

<u>Listen to your body</u>

Taking breaks throughout the day and during an activity is really important, but this does not mean you shouldn't be active. Instead, try to use the approach 'little and often' and rest before you are too tired to avoid long recovery times. Learn what triggers your fatigue and the early signs of when to take a break. Some people find the analogy of a traffic light helpful here.

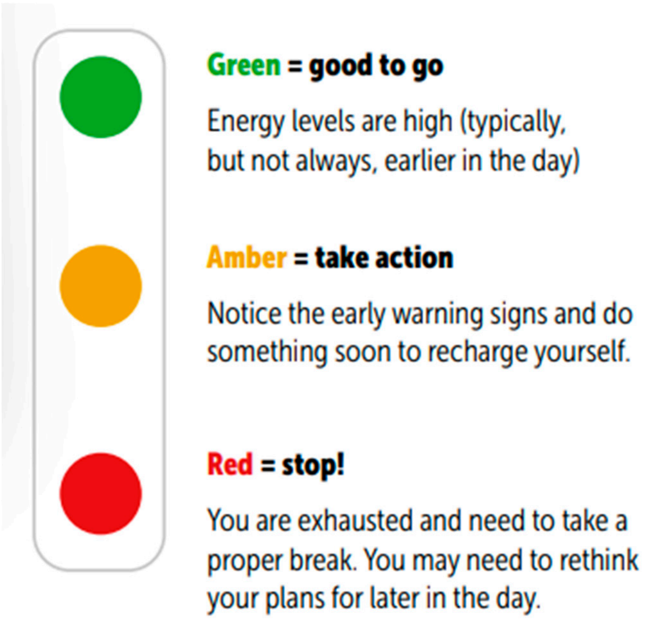

Plan ahead

Work out which activities drain you quickly and which gradually use your energy over time—plan for activities that need more energy at times during the day when you have more reserves. Avoid a 'boom then bust' approach. Also, plan for rest before and after the activity so you are not doing too much during the day. Routines and structure will help you learn what works well for you and plan your day so there will be time for a balanced and healthy lifestyle, including rest and activities.

Pace yourself - work out ways of 'recharging your battery' during the day

Once your triggers are understood, find ways to keep energy levels on a more even keel. This might be to sleep or rest for a while, but it may also be something like taking a short walk outdoors, doing a meditation or mindfulness exercise, or listening to a favourite piece of music. You may use an alarm or timer to help you remember to take a break.

Please be aware that after a cardiac arrest your sleep may be disrupted – this is also true for family members. A regular sleep routine, avoiding caffeine/alcohol/nicotine in the evening and avoiding long naps during the day might help. If you find that your sleep does not get better with these strategies, consider talking to your doctor.

Prioritise – work out which activities are truly necessary

Some daily activities are required, but others aren't. To find out which of yours are necessary, you could ask yourself: What do I need/want to do today? What can be put off until another day? What can I ask someone else to do for me?

Use strategies to support thinking skills

- Removing distractions (e.g., turning off background noise), writing things down (e.g., plans for the day, lists, recipes), and setting alarms as reminders can all decrease our mental demands and save energy – which may help us to pay attention, remember, stay on track, and achieve things more successfully.
- Also, remember that good lighting, limited background noise, a comfortable temperature, comfortable clothing, a calm (not cluttered) environment, and supportive seating can all make a huge difference. If you experience a physical type of fatigue, energy-saving techniques and aids may be helpful, such as sitting down while doing an activity or having devices/tools you often use stored close to your working space.

Look after yourself

- This includes (but is not limited to) eating healthily and regularly, doing regular physical exercise, and getting adequate sleep. Having a shared understanding with family, friends, and work colleagues about your feelings and what works for you is essential. Other people are more likely to understand if they know what you are experiencing.

- Also, remember to be kind to yourself! Try to manage levels of personal stress and worry by talking to others, using relaxation or meditation exercises, doing things you take pleasure in, and speaking to your GP if it becomes impossible to switch your head off from unhelpful or self-critical thoughts.

MEMORY AND COGNITIVE DIFFICULTIES AFTER A CARDIAC ARREST

During a cardiac arrest, the blood stops flowing through the body. This leads to an almost immediate loss of consciousness due to a lack of oxygen and nutrients in the brain.

People that survive and awake from the coma are often initially confused. They may forget conversations soon after having them, repeat themselves regularly, and sometimes mix up or not recognise family members. This is common after a critical illness, is often due to delirium ('acute confusional state') and is not necessarily a sign of brain damage. For many survivors, this initial confusion will improve and mostly resolve within a few days.

Some survivors, however, may experience ongoing and persistent difficulties with memory and thinking skills in the weeks/months following hospital discharge (cognitive impairment). These problems are usually much less severe than the initial confusion and may only be noticed by the survivors and/or their families. They may be the result of a mild brain injury. However, it is important to note that being fatigued, anxious or very low in mood can also significantly affect memory and other cognitive functions; when these issues are addressed, cognition usually improves significantly. This is not always true if a brain injury occurred.

Following a cardiac arrest, the most common cognitive difficulties involve *memory, attention*, and what is known as '*executive functioning'* – a set of skills that help you get things done by planning, organising, and switching between tasks ("multi-tasking").

UNDERSTANDING AND COPING WITH MEMORY PROBLEMS

To understand memory problems, we first need to know how memory works. There are many different types of memory.

Most people remember their childhood school, first car, wedding day, etc. We call this long-term memory. These memories are well stored in our brains, and, by and large, they are very resistant to any brain injury.

After a cardiac arrest, forming and retrieving new memories may become more difficult. This is what most people think of as short-term memory problems. When we create new memories, there are different processes at play. Firstly, we need to pay attention to the information we need to remember; then, we need to learn (or 'encode') the information and store it somewhere in our brains. Later we need to be able to recall/retrieve it when we need it. The correct functioning of all these processes makes us able to remember a conversation, a shopping list, an appointment etc.

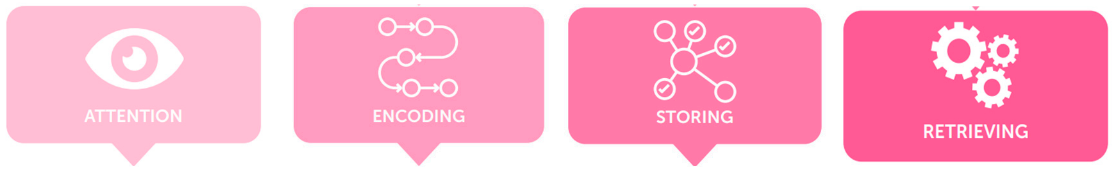

It is worth mentioning that no one's memory is perfect; some people may naturally be better at remembering conversations rather than images and songs or vice versa. No one has a perfect memory, and we all use internal and external strategies to support it (see below). If you experience more difficulties now in remembering things compared to before your cardiac arrest, you might find some benefit from increasing the use of these strategies.

Some people benefit from internal strategies, while others rely on external reminders. In general, cardiac arrest survivors with memory difficulties find it easier to use memory strategies they were already using before their event. However, learning a new skill with patience and perseverance is certainly possible. External strategies are often more helpful, especially if memory difficulties are more severe.

Internal strategies

Reducing distractions and maximising attention can be helpful - reducing all background noise, avoiding time pressure, and focusing only on one thing.

Repeating the same information several times will also help.

You can also try 'chunk' items together (for instance, in a shopping list, think of all the vegetables, all the drinks etc).

Creating an image of what you need to remember – or visualising – can also be helpful. •

External strategies

For most people, external strategies will be more helpful. These include using aids such as:

- Daily planners/calendars
- To-do lists
- Whiteboard and Post-it notes.
- Smartphones/tablets - alarms, voice recordings, calendars, and photographs of things to remember can all be used to support memory.
- Medication blister packs and dosette boxes.

HELPING A LOVED ONE

If your loved one has suffered a cardiac arrest, you may wonder how you can help them get better and back to a normal life. It is important to remember that life-threatening scenarios often affect the whole family somehow, you included. Perhaps even more so than the survivor, as you may have intervened in or witnessed the resuscitation.

Many survivors will recover from the initial period of confusion very quickly and completely. Some, however, may experience more long-term difficulties if they suffered a brain injury, which sometimes can be mild/very mild, but still impact day-to-day life. Depending on their circumstances and difficulties, they may need some help using the memory strategies (listed above), developing a routine, and ensuring they manage their fatigue as much as possible. Breaking down a task into smaller steps and avoiding overstimulation can also help to maximise a survivor's skills and abilities. Be aware that there will be 'good days' and 'bad days' – this will depend on many factors, some of which are beyond your control.

MENTAL HEALTH and WELL-BEING

Feeling worried and sad is natural and normal, particularly during a change or following a traumatic life event.

After leaving the hospital, many survivors describe going through a ‘rollercoaster’ of emotions; the initial joy and gratitude for being alive can sometimes turn into frustration around cognitive and physical limitations and concerns about returning to work and daily life.

Anxiety around experiencing another cardiac arrest is also common, as are anger and low mood, especially in the first few months. However, everyone’s experience is unique.

Family members can also go through their own recovery process, from the shock of witnessing the event to dealing with the transition from hospital to home. This can cause significant anxiety and can make a good recovery challenging.

LOW MOOD and DEPRESSION

Low mood is very common in the first few weeks or months but usually improves over time as survivors process what happened, gather more information, and increase their confidence to return to normal activities.

*There is no set timeframe for a 'return to normal'. H*owever, if a low mood continues for several weeks and significantly impacts life, it may be a sign of depression. Depression is a common mental health difficulty that includes low mood, withdrawal from social relationships, and less enjoyment in activities you usually find pleasurable. It can last for many weeks or longer.

When experiencing an episode of depression, a person will experience some changes in the way they think, behave, and feel, both physically and mentally. These changes are connected and are often described as a ‘vicious cycle’.

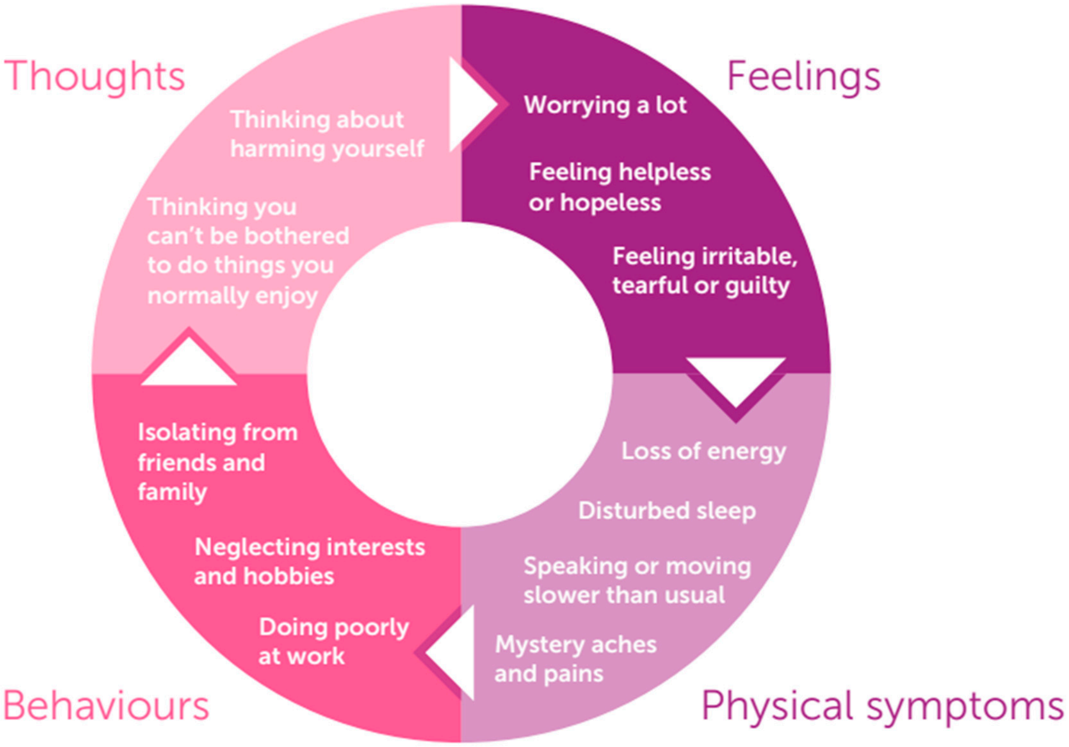

Self-help tips:

- Stay in touch!

Don't withdraw from life. Stay in touch with friends and family so you can talk to someone when you are feeling low.

- Keep active.

Do some form of physical exercise, even if it may feel challenging. Something as simple as a 10-20 minutes' walk can be very effective.

- Don't avoid things you find difficult.

Slowly and carefully face up to your fears. Ask someone to help and support you if you find it difficult.

- Eat healthily and don't drink too much alcohol.
- Have a routine.

Get up at your usual time and stick to a routine as much as possible.

….and seek help if you are still struggling!

-

ANXIETY AND STRESS

It is natural and normal to feel stressed. Many cardiac arrest survivors worry about their health and aches and pains; most also wonder what activities are safe now and what the future may hold.

It can be helpful to make a list of these questions and take them to your doctor/therapists: for instance, "What **symptoms should I be concerned about**?" "**What activities can I return to**?" "**What treatments are planned to reduce my risk**?" These can go a long way in helping with some of these worries.

Anxiety is like stress and worry, but on a regular, everyday basis, you might feel as if you cannot stop or control how much you worry about things. Research has shown that after a sudden cardiac arrest, around 1 in 4 survivors experience anxiety; this is also true for family members, especially if they have witnessed the arrest, performed CPR, or spent time in intensive care with their loved one during the most challenging days. Anxiety can affect the way we think and behave, similarly to depression. And again, all these changes are interconnected.

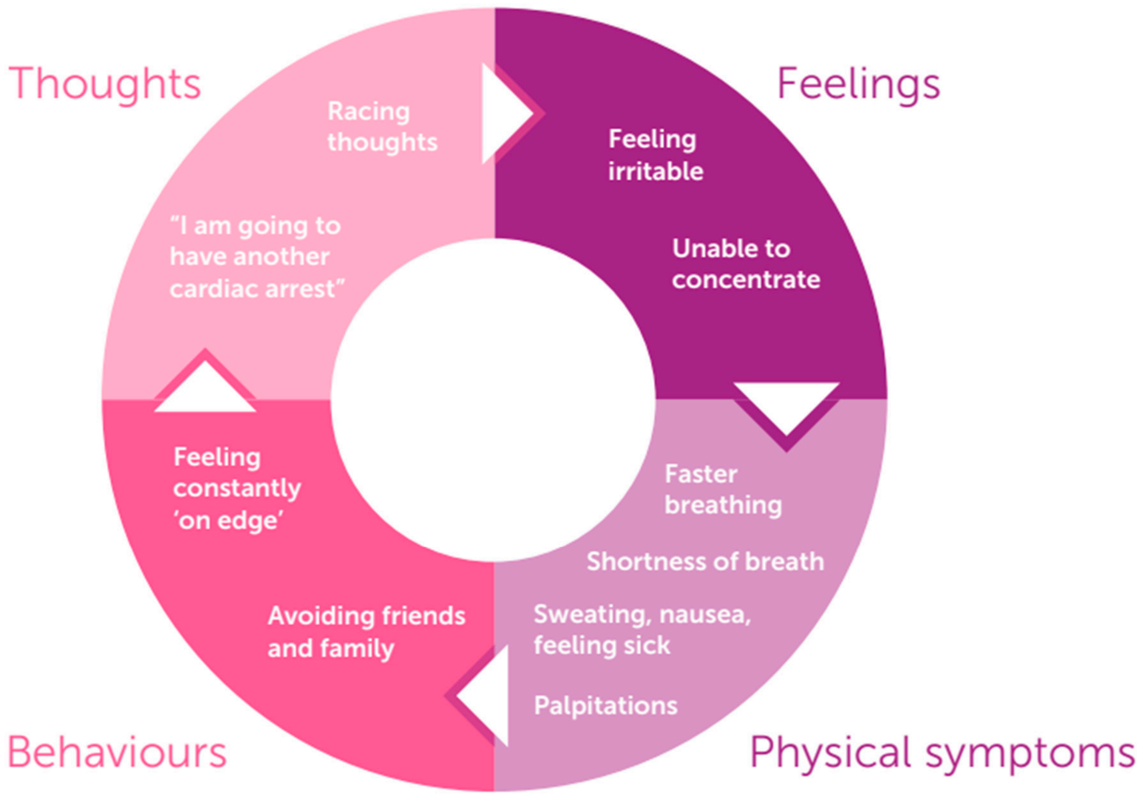

Post-Traumatic stress disorder (PTSD)

Some family members and survivors may struggle with nightmares, flashback and anxiety surrounding the circumstances of the cardiac arrest. They may also find sleeping or concentrating much more difficult. These symptoms can be a sign of post-traumatic stress disorder (PTSD). This can happen immediately after the traumatic event, or a few weeks/months after, but not everyone will feel like that. If you find you are experiencing these symptoms, please let your doctors know as specialist help is available.

Self-help tips for anxiety:

- Remember, you are not alone

Most people feel anxious at some point in life!

- Set small, achievable targets

Don’t try to do everything at once.

- Don’t avoid situations that make you anxious

Build up your tolerance little by little, if necessary. Ask someone to join you for support if needed.

- Find out how to get to sleep if you are struggling to sleep
- Try not to use alcohol, gambling, or drugs to feel better

These will only contribute to even poorer mental health.

- Try discussing your anxieties/feelings

with a friend, family member or healthcare professional.

- Eat a healthy diet.
- Exercise if you can!

Walking, running, swimming and yoga are examples of activities that can especially help you relax.

….and seek help if you are still struggling!
